# Assessment of the AQUIOS flow cytometer – An automated sample preparation system for CD4 lymphocyte PanLeucogating enumeration

**DOI:** 10.4102/ajlm.v8i1.804

**Published:** 2019-12-05

**Authors:** Daniel Rhodes, Guislaine Carcelain, Mike Keeney, Christophe Parizot, Dominika Benjamins, Laurine Genesta, Jin Zhang, Justin Rohrbach, Denise Lawrie, Deborah K. Glencross

**Affiliations:** 1 Clinical Affairs, Beckman Coulter Immunotech, Marseille, France; 2 Immunology Laboratory, Assistance Publique Hopitaux De Paris, Paris, France; 3 Lawson Health Research Institute, London Health Sciences Centre and St. Joseph’s Health Care, Victoria Hospital, London, Ontario, Canada; 4 Department of Immunology, University Hospital, Paris, France; 5 London Health Sciences Centre, London, Ontario, Canada; 6 Biomnis Laboratory, Lyon, France; 7 Life Science Flow Cytometry, Beckman Coulter Incorporated, Miami, Florida, United States; 8 Clinical affairs, Beckman Coulter Incorporated, Miami, Florida, United States; 9 National Health Laboratory Service, Charlotte Maxeke Johannesburg Academic Hospital, Johannesburg, South Africa; 10 Faculty of Health Sciences, University of the Witwatersrand, Johannesburg, South Africa; 11 National Health Laboratory Services, Johannesburg, South Africa

**Keywords:** HIV, cluster of differentiation 4, CD4 Enumeration, PanLeucogating

## Abstract

**Background:**

Flow cytometry has been the approach of choice for enumerating and documenting CD4-cell decline in HIV monitoring. Beckman Coulter has developed a single platform test for CD4+ T-cell lymphocyte count and percentage using PanLeucogating (PLG) technology on the automated AQUIOS flow cytometer (AQUIOS PLG).

**Objectives:**

This study compared the performance of AQUIOS PLG with the Flowcare PLG method and performed a reference interval for comparison with those previously published.

**Methods:**

The study was conducted between November 2014 and March 2015 at 5 different centres located in Canada; Paris, France; Lyon, France; the United States; and South Africa. Two-hundred and forty samples from HIV-positive adult and paediatric patients were used to compare the performances of AQUIOS PLG and Flowcare PLG on a FC500 flow cytometer (Flowcare PLG) in determining CD4+ absolute count and percentage. A reference interval was determined using 155 samples from healthy, non-HIV adults. Workflow was investigated testing 440 samples over 5 days.

**Results:**

Mean absolute and relative count bias between AQUIOS PLG and Flowcare PLG was −41 cells/*µ*L and −7.8%. Upward and downward misclassification at various CD4 thresholds was ≤ 2.4% and ≤ 11.1%. The 95% reference interval (2.5th – 97.5th) for the CD4+ count was 453–1534 cells/*µ*L and the percentage was 30.5% – 63.4%. The workflow showed an average number of HIV samples tested as 17.5 per hour or 122.5 per 8-hour shift for one technician, including passing quality controls.

**Conclusion:**

The AQUIOS PLG merges desirable aspects from conventional flow cytometer systems (high throughput, precision and accuracy, external quality assessment compatibility) with low technical operating skill requirements for automated, single platform systems.

## Introduction

Flow cytometry has been the system of choice for CD4 lymphocyte enumeration and documentation of the decline of CD4 T-cells associated with immunosuppression and lowered counts in HIV-positive patients.^1,2,3^ Many diverse CD4 systems that offer solutions to improve and ensure the quality of testing and improve access to testing technologies have been described over the last 25 years.^4,5^

In resource-limited settings, there are many instances where laboratory infrastructure is a limiting factor. However, flow cytometric systems and simpler technologies (such as point-of-care technologies), when used in a tiered laboratory approach, can offer a solution.^6,7,8,9,10^ In such an approach, primary centres offer simplified testing and refer testing for flow cytometry analysis to secondary or tertiary centres.^11,12,13^ Despite the relative technical complexity, flow cytometry systems, particularly those that require less technical expertise, have been implemented with success in some national programmes.^11,12^ The suitability of proposed instrumentation must be assessed in the context of the destination laboratory. Concerns such as the level of technical skill required for operation (ease of use, training, and automation), daily sample load and turn-around time requirements, external quality assessment programme compatibility and quality control reagent availability, supplier availability and support, transit requirements, infrastructure, and cost per test should be considered.^11,14^

The PanLeucogating (PLG) CD4 counting method^12,15,16^ incorporates a simple gating strategy with only CD45 and CD4 to enumerate CD4 lymphocytes. Quality assessment programmes reported improved performance of PLG CD4 counting and revealed better quality in both the intra- and inter-laboratory reported percent coefficient of variation outcomes.^12,17^ Decreased costs of the simplified system also played an important role in addressing some of the aforementioned concerns.^16,18^ This method was adopted as the predicate method by the South African National Health Laboratory Service (NHLS) in 2004. The NHLS programme had grown to 35 laboratories by 2007,^12^ reaching 60 networked CD4 laboratories by 2014^11^ in a tiered system utilising either Beckman Coulter FC500 (Beckman Coulter, Inc., Miami, Florida, United States) or XL (Beckman Coulter, Inc., Miami, Florida, United States) instruments according to service workload requirements.^11^

A single platform volumetric flow cytometer (AQUIOS, Beckman Coulter, Inc., Miami, Florida, United States) was recently developed that utilises a conventional CD4 gating method based on CD45 and CD3 with both CD4 and CD8 for CD4-positive and CD8-positive T-cell lymphocyte counts and percentages. This system was updated in 2013-2014 using bead-based counting for use with the current South African laboratory network PLG predicate^13,14^. The AQUIOS system is fully automated from sample preparation to flow cytometry analysis. It allows for operator independent loading and testing for multiple samples. It has pre-configured panels or protocols that are not modifiable by the user, enabling standardised testing. In line with the tiered model adopted by the National Health Laboratory Service, PLG testing on the AQUIOS system (AQUIOS PLG) was proposed as the system to replace aging and redundant FC500 and XL flow cytometers operational within the South African network, as well as extend its use into small laboratories that offered basic clinical pathology but no CD4 services.^19^

The objective of this study was to compare the performance of AQUIOS PLG with traditional PLG CD4 methods generated on the FC500 instrument at a local South African site, as well as established CD4 reference centre sites in high-income countries. Additionally, normal samples were collected and these data were used to calculate a reference interval to establish whether normal counts generated by AQUIOS PLG matched other published reference intervals.^20,21,22,23,24,25,26,27,28,29,30,31,32,33,34,35,36,37,38,39,40,41,42^

## Methods

### Ethical considerations

All sites had ethics committee or internal review board approval for the collection of samples or use of leftover samples and use of minimal demographic data of age and gender or a waiver was in place for use of these samples. Use of leftover samples for research purposes was agreed to at the time of routine laboratory blood draw. The following ethical clearances were in place: South Africa (identification number: M121020); France (Lyon; identification number: AC-2013-1808); Canada (identification number: 09763E); and the United States (identification number: 10259-05). In France (Paris), consent was given at the time of routine blood draw for the use of leftover samples for research under a waiver according to current French legislation (Loi Jardé, n°2012-300). Patients providing samples for the reference interval study signed a consent form.

### Specimens

#### Method comparison

Patient samples were obtained from incoming routine laboratory specimens for CD4 testing between November 2014 and March 2015 from four different centres located in Canada; Paris, France; Lyon, France and South Africa. A total of 270 samples from HIV-positive adult and paediatric patients were tested, ranging in age from 2 months to 77 years. Thirty samples (22 adults, 8 children) were excluded for the following reasons: CD4 < 20 cells/*µ*L (the instrument’s lower limit of quantitation) (18 samples), operator error with manual FlowCount addition (FC500) (4 samples), short blood draw (2 samples), reliability quality control failure (FC500) (2 samples), clot (1 sample), clog (1 sample), insufficient lymphocytes (1 sample) and duplicate patient (1 sample). Testing was performed in duplicate, with replicate 1 used for analysis, except for two samples, for which replicate 2 was used due to system error for replicate 1 (1 high count rate and 1 clog). Two hundred and forty samples were included in the final analysis with specimen ages at time of testing ranging from 20 to 71 hours from collection.

#### Reference interval in healthy adults

Participants were enrolled between December 2014 and February 2015 from three centres located in Canada; Paris, France and the United States. Samples were obtained from: 1) healthy, non-HIV patient donors who were either hospitalised patients or outpatients with no haematological disease upon final diagnosis (Canada); 2) leftover samples from healthy, non-HIV volunteers donating blood to the Etablissement Francais du Sang (Paris, France); and 3) self-reported healthy, non-HIV participants enrolled through the internal donor programme at Beckman Coulter (Miami, Florida, United States). Participants with CD4 < 300 cells/*µ*L were excluded so as not to include participants with potential idiopathic CD4+ lymphopenia.^20,21^ A total of 173 participant samples from healthy adults aged 18–65 years, with normal complete blood counts and differential were tested. Eighteen samples were excluded for the following reasons: haematologic diagnosis (10 samples), lymphopenia (3 samples), participants < 18 years of age (2 samples), lymphocytosis (1 sample), time of collection missing (1 sample) and CD4 < 300 cells/*µ*L (1 sample). One hundred and fifty-five samples were included in the final analysis, all tested within 24 hours of collection.

#### Workflow

Samples were obtained from incoming routine laboratory samples for CD4 testing at one centre in South Africa over five days in February 2015. A total of 440 participant samples were tested over five days.

### Laboratory

**FIGURE 1:**
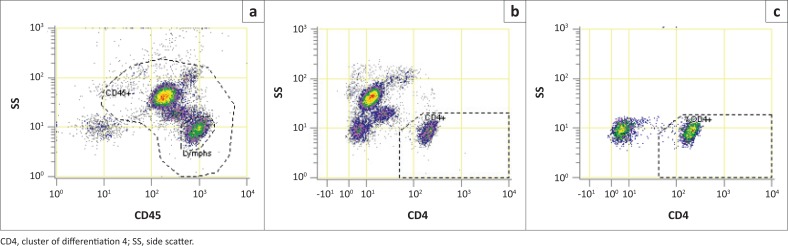
CD4 T-cell enumeration by Panleucogating with AQUIOS Flow cytometer, Canada; Paris, France; Lyon, France; and South Africa, November 2014 to March 2015. (a) CD45 versus side scatter plot is used to identify total leukocytes (CD45-positive); (b) All events gated in the CD45-positive region (Pan-Leucogate) are used to plot CD4 versus side scatter to identify CD4-positive lymph cells (CD4-positive Count/μL). (c) Lymphs gate from A is used to plot the CD4 versus side scatter to calculate the CD4 percentage of lymphoid cells (CD4-positive Lymph percent).

Prior to each day’s testing, stabilised blood products IMMUNO-TROL Cells (normal CD4 count) (Beckman Coulter, Inc., Miami, Florida, United States) and IMMUNO-TROL Low Cells (Beckman Coulter, Inc., Miami, Florida, United States) passed their assay requirements as quality control material. Samples were collected into ethylenediaminetetraacetic acid vacutainers and tested in duplicate on both instruments for method comparison and only on AQUIOS for reference interval. The PLG gating strategy, used for both instruments, is detailed in Figure 1. All reagents were supplied by Beckman Coulter, Inc., Hialeah, Florida, United States and Immunotech, Marseille, France.

#### AQUIOS instrument

CD4+ counts and percentages were determined using AQUIOS PLG, as per manufacturer’s instructions for use. Each site had an instrument and all required reagents.

#### FC-500 instrument

Comparator testing was done using FlowCARE PLG CD4 Reagent on the Beckman Coulter FC-500 MCL Flow Cytometer (Flowcare PLG) per the manufacturer’s instructions for use. All required reagents were provided. A TQ-Prep™ Workstation (Beckman Coulter, Inc., Miami, Florida, United States) was used for red cell lysing. Sites either used a PrepPlus™ 2 Workstation (Beckman Coulter, Inc., Miami, Florida, United States) for specimen preparation or manual preparation in place of instrumentation.

### Statistical analysis

Clinical and Laboratory Standards Institute guidelines EP09-A2 and EP28-A3^43,44^ for method comparison^43^ and reference interval determination^44^ were followed. Replicate 1 was used for analysis, with replicate 2 used for the resolution of replicate 1 discrepancies. For method comparison, the number and percentage for sex, adult or paediatric specimens, mean age, CD4 count and percentage and CD4 count range were calculated. AQUIOS PLG and Flowcare PLG results were analysed using Deming regression to estimate bias at the clinically relevant CD4-positive levels of 50, 100, 200, 350 and 500 cells/*µ*L. Weighted Deming regression was used for count because the variability (scatter) of the data depended on the range of measurements, while simple Deming regression was used for percentages. The coefficient of determination, *R*^2^ (Pearson correlation squared), was used to measure the overall correlation between the two methods. Bland-Altman analysis^45^ was used to calculate the mean and median difference between methods. Mean and median relative bias expressed as percent was also calculated. Mean and median absolute difference and relative difference were calculated by CD4 subgroups ≤ 200, 201–1000, and > 1000 cells/*µ*L and ≤ 350, 351–1000, and > 1000 cells/*µ*L, where 200 cells/*µ*L and 350 cells/*µ*L represent antiretroviral treatment (ART) thresholds and 1000 cells/*µ*L was used to control for variability of paediatric samples. Upward and downward misclassification probabilities at ART thresholds of 100, 200, 350 and 500 cells/*µ*L were determined (the method has been described elsewhere^46^). Upward misclassification represents the percentage of additional patients who would fall above a defined threshold with the new test, whereas downward misclassification represents the percentage of additional patients that would fall below this threshold. Mean percent similarity with a standard deviation (SD) and coefficient of variance was also calculated (the method has been described elsewhere^47^). For the reference interval, demographic characteristics were calculated (number and percentage for sex and mean ± SD for age). Mean, SD, median, and range for both CD4-positive count and CD4-positive percentage were calculated by sex and for total participants. The 95% (2.5th – 97.5th) reference interval was determined using non-parametric methods. Statistical differences in variables sex, adult or paediatric patient status were determined by *t*-test for the means and non-parametric Mann Whitney U test for the medians. For workflow analysis, time to first result, average sample results per hour, and sample results per 8-hour shift were calculated. Statistical analysis was performed using Microsoft Excel (Microsoft Corporation, Redmond, Washington, United States) with Analyse-IT.

## Results

### Method comparison

**FIGURE 2:**
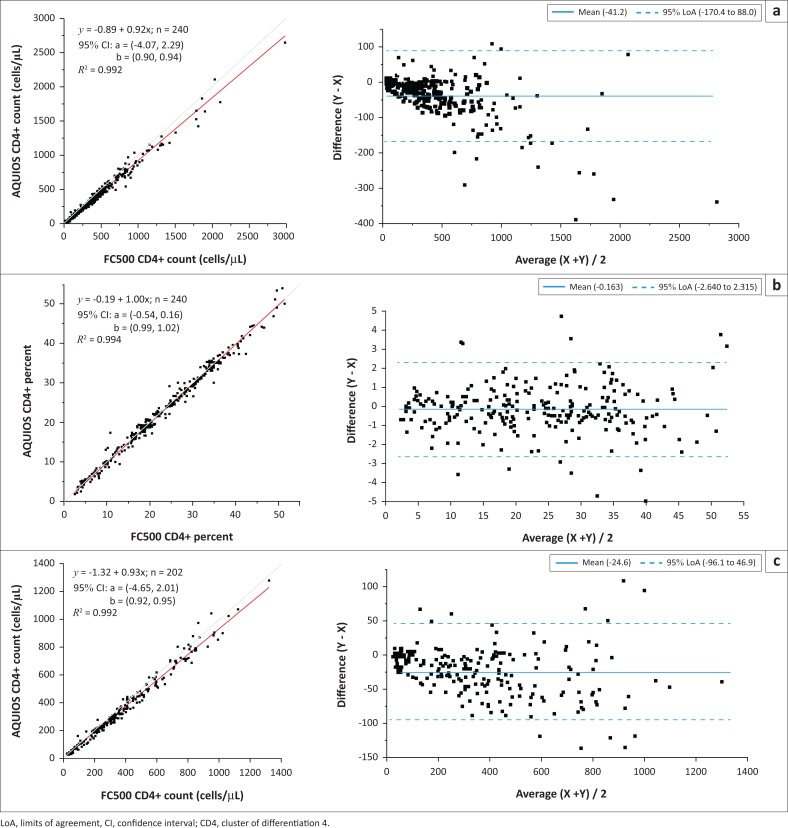
Deming regression and Bland-Altman for AQUIOS PanLeucogating versus Flowcare PanLeucogating, Canada; Paris, France; Lyon, France; and South Africa, November 2014 to March 2015. (a) Absolute CD4 count in cells/*μ*L for adults and children, (b) Percentage of CD4 for adults and children, (c) Absolute CD4 count in cells/*μ*L for adults only.

Two hundred and forty specimens were included in this method comparison study, 92 (38.7%) were obtained from female participants (mean age 35.2 years) and 146 (61.3%) were from male participants (mean age 42.6 years); 202 (84.2%) were adults and 38 (15.8%) were children. Mean CD4 count and percentage were not significantly different by sex (females: 339 cells/*µ*L and 22.65%, males: 359 cells/*µ*L and 23.47%; *p* > 0.05), but were by adult versus paediatric participants (adults: 361 cells/*µ*L and 22.20%, children: 948 cells/*µ*L and 32.28%; *p* < 0.001). The CD4 count range showed a higher minimum–maximum for paediatric specimens (46–2645 cells/*µ*L) than adult specimens (24–1278 cells/*µ*L). CD4 count for all specimens showed an *R*^2^ = 0.992 and mean bias of −41 cells/*µ*L between AQUIOS PLG and Flowcare PLG (Figure 2a). Mean relative bias was −7.8%. Of 12 samples with a CD4 count above 1250 cells/*µ*L, 11 were from paediatric participants. For CD4 percentage, *R*^2^ was 0.994 and there was an average bias of −0.16% (Figure 2b). Because the mean CD4 count between adults and children was significantly different, adult specimens were analysed separately, showing an *R*^2^ = 0.992 and average bias of −25 cells/*µ*L (Figure 2c). Mean relative bias was −6.8%.

Bias analysis by CD4 subgroups based on ART thresholds of 200 cells per *µ*L and 350 cells per *µ*L showed relatively consistent median relative bias for samples below and above the respective thresholds: −7.8% and −8.1%, and −7.9% and −8.1% (Table 1).

**TABLE 1:** CD4 count absolute and relative bias between AQUIOS PanLeucogating and Flowcare PanLeucogating overall, by CD4 subgroup and at clinically relevant CD4 levels, Canada; Paris, France; Lyon, France; and South Africa, November 2014 to March 2015.

CD4 count (cells/*µ*L)	*N*	Absolute difference			Relative difference†	
		Mean		Median (cells/*µ*L)	Mean (%)	Median (%)
		cells/*µ*L	95% CI			
**Overall**	240	−41	−50 – −33	−27	−7.8	−8.2
**Bias by subgroup**						
≤ 200	62	−7	−12 – −2	−5	−7.4	−7.8
201–1000	155	−39	−47 – −31	−35	−7.8	−8.1
> 1000	23	−145	−196 – −95	−133	−9.4	−11.4
≤ 350	107	−14	−18 – −10	−12	−7.8	−7.9
351–1000	110	−46	−57 – −35	−45	−7.5	−8.1
> 1000	23	−145	−196 – −95	−133	−9.4	−11.4
**Bias by level**						
50	-	−5	−8 – −2	-	−9.8	-
100	-	−9	−11 – −6	-	−8.9	-
200	-	−17	−20 – −14	-	−8.5	-
350	-	−29	−34 – −25	-	−8.3	-
500	-	−41	−48 – −34	-	−8.2	-

CD4, cluster of differentiation 4.

† , test – reference / (reference × 100).

Upward misclassification ranged from 0.0 to 2.4% and downward misclassification ranged from 1.5 to 11.1%, depending on the threshold (Table 2). Mean percent similarity (SD, coefficient of variance) was 96.1% (6.1%, 6.3%) for the absolute CD4 count and 99.5% (3.9%, 4.0%) for the CD4 percentage.

**TABLE 2:** Misclassification percentages at various CD4 count thresholds, Canada; Paris, France; Lyon, France; and South Africa, November 2014 to March 2015.

AQUIOS CD4 count threshold	All		South Africa (%)	Paris, France (%)	Canada (%)	Lyon, France (%)
	%	*n*/*N*				
**100 cells/*µ*L**						
Upward (%)	2.4	1/41	0.0	0.0	0.0	12.5
Downward (%)	1.5	3/199	2.2	0.0	0.0	3.7
**200 cells/*µ*L**						
Upward (%)	1.6	1/62	0.0	0.0	0.0	8.3
Downward (%)	3.9	7/178	1.2	4.9	3.1	13.0
**350 cells/*µ*L**						
Upward (%)	0.9	1/106	0.0	0.0	3.4	0.0
Downward (%)	8.2	11/134	8.7	18.2	5.0	0.0
**500 cells/*µ*L**						
Upward (%)	0.0	0/150	0.0	0.0	0.0	0.0
Downward (%)	11.1	10/90	12.7	0.0	7.7	22.2

CD4, cluster of differentiation 4.

### Reference interval in healthy adults

Demographic characteristics for the 155 samples included in the reference interval analysis showed the proportions of female participants were Paris, France, 0.73; Canada, 0.59; United States (0.39). The mean overall ages were 40 years (Canada); 45 years (United States); the mean female-to-male ages were 36 versus 45 years (Canada) and 52 versus 41 years (United States). Specific age data for samples from Paris, France were not available. The mean CD4-positive count ± SD was higher in Canada (1009 ± 239 cells/*µ*L) compared to both the United States (866 ± 221 cells/*µ*L) and Paris, France (802 ± 273 cells/*µ*L). Similar results were seen for the mean CD4-positive count by sex at each site. The 95% reference intervals for both absolute CD4-positive counts and percentages by site were similar and overlapped.

The mean CD4 absolute count and percentage for female and male participants were not different statistically (*p* = 0.61 and 0.48, respectively) (Table 3). The overall mean CD4 positive count ± SD was 888 ± 255 cells/*µ*L and mean CD4-positive percentage ± SD was 46.82 ± 7.86%. The 95% reference interval (2.5th – 97.5th) for CD4-positive count and percentage was 453–1534 cells/*µ*L and 30.5% – 63.4% (Table 4). Recent, previously established reference interval results for CD4 absolute count and percentage from healthy, non-HIV individuals from different parts of the world using differing instrument platforms show consistent results (Table 5).

**TABLE 3:** Demographic characteristics and means, medians, and ranges for CD4 and percentages for a healthy adult reference interval, Canada; Paris, France; and United States, December 2014 to February 2015.

Measurement	Sex		*p*	Overall
	Female	Male		
**Demographics**				
*N*	86	69	-	155
Percentage	55.5	44.5	-	-
Age†	43.5	42.2	0.58	42.8
SD	13.0	13.3	-	13.1†
**CD4-positive count (cells/*µ*L)**				
Mean	879	900	0.61	888
SD	267	241	-	255
Median	874	897	0.61	878
Range (min-max)	352–1573	456–1778	-	352–1778
**CD4-positive percentage**				
Mean	47.25	46.28	0.45	46.82
SD	7.67	8.12	-	7.86
Median	47.12	46.29	0.48	46.83
Range (min-max)	28.05–69.07	27.90–63.84	-	27.90–69.07

CD4, cluster of differentiation 4; SD, standard deviation.

† , Based on *N* = 107 (age other than between 18 and 60 years not provided by Paris, France site).

**TABLE 4:** 95% reference interval (2.5th – 97.5th) for apparently healthy adults, Canada; Paris, France; and United States, December 2014 to February 2015.

Overall	CD4 %		CD4 cells/*µ*L	
	Value	90% confidence interval	Value	90% confidence interval
Lower reference interval	30.47	27.90–35.13	453	352–506
Upper reference interval	63.38	60.11–69.07	1534	1329–1778

CD4, cluster of differentiation 4.

**TABLE 5:** Comparison of overall normal reference intervals (2.5th – 97.5th) for CD4 lymphocytes in HIV-negative adults, Canada; Paris, France; Lyon, France; and South Africa, November 2014 to March 2015.

Region	Study	Technology/platform	*N*	Age (years)	Sex (% female)	CD4-positive absolute count		CD4-positive percentage 95% Ref Int
						Mean (cells/*µ*L)	95% Ref Int	
**United States/Canada/Europe**	This study 2015	AQUIOS/single†	155	18–65	55.5	888	453–1534	30.5–63.4
	AQUIOS Tetra 1 2013^20^	AQUIOS/Single	161	18–65	47.8	904	518–1472	33.6–64.8
	Germany 2005^21^	FACSCalibur/dual†	100	19–84	50.0	870‡	490–1640	30.0–59.0
	Italy 1999^22^	multiple	968	18–70	45.0	940	493–1666	32.0–61.0
**Latin America**	Mexico City 2013^23^	FACSCount/single	400	20–40	50.0	800	340–1260	NA
**Southern Africa**	South Africa 2009^24^	EPICS-XL/single†	675	18–55	87.3	1104	548–2045	29.8–58.1
	Botswana 2004^25^	FACSCount/single	437	Adults	32.7	759	366–1318	NA
**Eastern Africa**	Malawi 2011^26^	FACSCalibur/single	214	Adults	50.5	863	276–1730	NA
	Tanzania 2009^27^	FACSCount/dual	102	> 10	58.8	746	312–1368	NA
	Tanzania 2008^28^	FACSCalibur/single	273	19–48	47.6	802‡	406–1392	27–52
	Tanzania 2003^29^	MultiSET/single	214	17–61	50.0	843	405–1500	27.0–55.0
		SimulSET/dual	214	17–61	50.0	853	403–1604	23.1–54.0
	Kenya 2013^30^	FACSCalibur/single	315	16–60	27.0	920	343–1493	24.0–48.0
	Kenya 2008^31^	FACSCalibur/dual	1293	18–55	34.4	851	421–1550	30.0–55.0
	Eastern Africa 2009^32^	Multiple FACS/dual	2100	18–59	48.6	860‡	457–1628	NA
	Uganda 2011^33^	EPICS-XL/dual†	172	15–70	43.6	938	418–2105	18.8–54.1
	Ethiopia 2014^34^	EPICS-XL/dual	320	18–64	49.7	820	321–1389	NA
	Ethiopia 1999^35^	FACScan/dual	142	15–45	35.2	775	366–1235	NA
**Western Africa**	Nigeria 2009^36^	Cyflow/single	2570	> 18	47.0	847	365–1571	NA
	Burkina Faso 2007^37^	FACSScan/single	186	18–78	47.8	1082‡	631–1696	30.0–53.0
**Indian Subcontinent**	Chennai 2009^38^	FACSCount/dual	213	18–56	39.4	926	376–1476	21–59
	India 2003^39^	EPICS-XL/dual	94	18–74	41.5	865	430–1740	30.8–49.6
**Asia/Southeast Asia**	Singapore 2004^40^	FACSCalibur/single	232	16–65	55.2	838	401–1451	23.0–48.2
	Hong Kong 2013^41^	FC500/single	273	17–59	45.0	760	396–1309	28.1–53.4
	Shanghai 2004^42^	Bryte-HS/dual	614	16–50	38.6	727	415–1189	NA

Ref Int, Reference Interval; NA, not applicable.

† , PanLeucogating used.

‡ , Median.

As per protocol analysis that is presented in the AQUIOS PLG test instructions for use, six participant samples from Paris were excluded. These samples had CD4 levels of 300–500 cell/*µ*L. As per protocol, samples with CD4 levels < 500 cells/*µ*L required an in-house medical monitor review of the complete blood count with differential to confirm haematological normal status and qualify for inclusion. The complete blood count with differential results were not provided for samples from the French site, so for the per protocol analysis, those with CD4 < 500 cells/*µ*L were excluded, as it was not possible to exclude the presence of idiopathic CD4-positive T-lymphocytopenia. For this manuscript analysis, these samples with a CD4 ≥ 300 −500 cells/*µ*L were included for the following reasons: 1) the French samples had a confirmed haematologically normal complete blood count with differential performed at the Etablissement Francais du Sang (although results were not available to Beckman), and 2) the Centers for Disease Control does not consider a decreased CD4 level in healthy, non-HIV patients significant unless < 300 cells/*µ*L.^17,18^ As expected, the inclusion of these samples decreased the lower reference interval value from 532 cells/*µ*L to 453 cells/*µ*L. The upper reference interval value was not affected and thus remained unchanged.

### Workflow

A total of 440 samples were tested over 5 days (Table 6). The average time to the first result was 39.2 minutes. The average number of samples processed per hour was 17.5 or 122.5 for an 8-hour shift, minus 1 hour for start-up, quality control testing and shut-down. Technician hands-on time required one hour, including start-up, quality control testing, sample testing and shut-down.

**TABLE 6:** High volume workflow results with AQUIOS PanLeucogating, South Africa, February 2015.

Testing day	No. of samples tested	Time to first result (minutes)	Time from start of first sample to result for last sample (hours:minutes)	Samples/h	Samples/8 h shift†
1	105	38	5:50	18.0	126.0
2	86	38	5:09	16.7	116.9
3	72	47	4:26	16.2	113.4
4	90	35	4:39	19.4	135.8
5	87	38	5:07	17.0	119.0

**Overall**	**440**	**39.2**	**25:11**	**17.5**	**122.5**

h, hour.

† , Includes 1 hour for start-up, quality control, and shut-down.

## Discussion

In this study, we compared PLG on the AQUIOS Flow Cytometer versus PLG CD4 counts generated by FC500 instruments. Additionally, CD4 counts generated from normal individuals were used to establish and gain insights into the reference interval of the CD4 counts of patients tested by AQUIOS PLG with respect to other published reference intervals.

The method comparison of AQUIOS PLG to Flowcare PLG showed a mean absolute count bias of −41 cells/*µ*L and a mean relative bias of −7.8% including both adult and paediatric HIV samples. For adults only, a mean absolute count bias of −25 cells/*µ*L with a mean relative bias of −6.8% was observed, similar to outcomes noted in separate evaluations.^48^ The slight negative bias in our study appears to be largely platform related and not related to the gating strategy used. Where different gating strategies (AQUIOS Tetra, with primary CD45 bright and CD3 gating to define CD4 lymphocytes and AQUIOS PLG which relies only on CD45 total and CD4/SS to discriminate monocytes) were applied on the same platform, a bias of just 10 cells/*µ*L with an *R*^2^ of 0.996 (unpublished data) was noted. This finding is similar to the slight positive bias previously reported where bead-based counting versus volumetric-based counting comparison was performed.^6^ While the mean and median absolute count difference increases with the CD4 level, the relative difference remained stable (around 8% – 11%) across the entire range of samples tested, even at counts above 1000 cells/*µ*L (where clinical importance is less). Paediatric patients are known to have higher CD4 absolute levels and higher variability in these counts than adults,^49^ and this was seen in our study as well, where mean and maximum CD4-positive counts were higher for paediatrics than adults.

Misclassification probability measures are used to determine the likelihood that a patient’s result for a new test compared to a reference test will be classified above or below a defined threshold used in clinical decision-making^50,51^ These measures provide a more direct interpretation for health policy and management decision making with regard to potential financial and healthcare impacts from implementation of new instrumentation.^51^ Misclassification results for CD4 should be interpreted for regions where World Health Organization guidelines^52^ for universal testing and treatment are not applied and where varying treatment thresholds still exist. Our results indicate that the introduction of AQUIOS PLG may result in ≤ 2.4% upward and ≤ 11.1% downward misclassification.

The CD4-positive absolute count mean and 95% (2.5th – 97.5th) reference interval among healthy, non-HIV adults from our study were consistent with previously established reference intervals in other studies^20,21,22,23,24,25,26,27,28,29,30,31,32,33,34,35,36,37,38,39,40,41,42^ on healthy individuals from different parts of the world (Table 5). This consistency of reference intervals established the equivalency of PLG gating methods in the context of reference intervals where typically conventional gating strategies were used. Reference intervals enable meaningful interpretation of a patient’s laboratory results. It is recommended that laboratories establish local reference ranges for clinical use^44,53^ due to known differences relating to location, race or ethnicity, sex, age, disease burden and drug intake.^21,22,27,28,29,33,36,38,39,42,49,54^ In many countries, especially low- and middle-income countries, resources frequently limit local reference range development^24^ and there are few published reference interval datasets. Interpretation of reporting is largely based on reference ranges published from high-income, industrialised areas of North America and Europe.^24^ Our results, however, indicate that CD4 results across multiple geographies, instruments and platforms are quite consistent, including results for CD4 obtained by PLG on the AQUIOS.

The circumstances and conditions for CD4 testing in laboratories vary in low- and middle-income countries. Challenges faced vary and include a basic lack of infrastructure such as an unstable electricity supply, a lack of cold storage facilities to a paucity of skills.^55^ The burden of HIV disease may also frequently dictate high workload volumes in certain countries.^11,12^ Manual sample preparation requires multiple pipetting, which in turn increases pipetting errors. However, automated sample preparation lowers percent coefficients of variance through the reduction of human pipetting errors.^12,56^ The simplified PLG CD4 method^12,16^ is the predicate system of the South African programme, and it combines the reliable bead-based testing technology^8^ and a gating strategy.^8,12,16,57^ PLG on the AQUIOS platform is regarded as a suitable candidate for this programme to replace older aging equipment currently used^12,48^ It is envisaged that its user-independent and on-board sample preparation features could improve testing outcomes in small laboratory sites with fewer staff or less technical flow cytometry expertise. Thus, this enables them to provide local CD4 services even with workload equivalents of up to 100 samples per day and improve local service delivery turn-around times in more remote parts of South Africa.^11,19^

The workflow showed a medium-high throughput level. Lower throughput (12 samples per hour or 96 samples per shift tested) was seen in a previous workflow study performed in the same laboratory using manual preparation and analysis on a FC-500 flow cytometer, and, 6 hours of hands-on technician time were required.^58^ The AQUIOS PLG daily workflow includes available quality control material and the system works with stabilised blood products, making it compatible with external quality assessment programmes. This allows for ongoing monitoring of intra- and inter-laboratory precision which is important for quality management of large-scale country-wide programmes.^11,12^ The system tracks quality control results and alerts user and technical support staff of deviations or trending. As a single platform system with on-board sample preparation, no additional laboratory equipment was needed.

Lastly, it is important to briefly discuss the relevance of CD4 counting in the face of recent World Health Organization guidelines^52^ which recommend that all patients who are HIV-positive start ART, irrespective of their CD4 counts. For the past 30 years, medical personnel caring for HIV patients have used CD4 counts as prognostic indicators of disease progress^3^ or death.^2^ Also, the CD4 count is used to determine eligibility for initiating ART, managing and treating opportunistic infections and monitoring the patient’s response to ART.^59^ It is widely agreed that HIV viral load testing is the optimal assay to monitor the response to ART or determine treatment failure.^60,61^ However, in light of the documented worldwide number of individuals with advanced HIV disease^61,62,63^ and lack of funding and infrastructure for routine viral load testing in low- to middle-income geographies, CD4 counts will continue to play an important role in managing HIV patients in terms of: stratifying long-term risk, fast-tracking onto ART outside the standard of care^64^ and identifying patients with immunological or clinical failure.^60,65,66^ The high number of individuals with advanced HIV disease also dictates that CD4 counts should play an important role in identifying patients at risk of opportunistic infections, such as cryptococcal disease, prophylactic treatment for tuberculosis and *Pneumocystis* pneumonia.

### Limitations

Our study utilised samples mainly from high-income, industrialised or urban areas, and such as may not completely represent samples found in an entirely African population.

### Conclusion

The AQUIOS PLG merges desirable aspects from conventional flow cytometer systems (high throughput, precision and accuracy, external quality assessment compatibility) with low technical skill requirements for automated, single platform systems.

## Trustworthiness

The findings of these studies should be used per the scope of the study and with regard to the indicated limitations. The results review and release on AQUIOS should be performed by a qualified professional.

## References

[1] Prince HE, Lesar WJ Simultaneous determination of absolute total lymphocyte and CD4+ lymphocyte levels in peripheral blood by flow cytometry. Am J Clin Pathol. 1989;92(2):206–209. 10.1093/ajcp/92.2.206 2569266

[2] Pedersen C, Gerstoft J, Tauris P, et al Trends in survival of Danish AIDS patients from 1981 to 1989. AIDS. 1990;4(11):1111–1116. 10.1097/00002030-199011000-00009 1980821

[3] Lee CA, Phillips AN, Elford J, Janossy G, Griffiths P, Kernoff P Progression of HIV disease in a haemophilic cohort followed for 11 years and the effect of treatment. BMJ. 1991;303(6810):1093–1096. 10.1136/bmj.303.6810.1093 1781870 PMC1671314

[4] Mandy F, Janossy G, Bergeron M, Pilon R, Faucher S Affordable CD4 T-cell enumeration for resource-limited regions: A status report for 2008. Cytometry B Clin Cytom. 2008;74 Suppl 1:S27–S39. 10.1002/cyto.b.20414 18307251

[5] Mandy F, Nicholson J, Autran B, Janossy G T-cell subset counting and the fight against AIDS: Reflections over a 20-year struggle. Cytometry. 2002;50(2):39–45. 10.1002/cyto.10097 12116344

[6] Janossy G, Jani I, Gohde W Affordable CD4(+) T-cell counts on ‘single-platform’ flow cytometers I. Primary CD4 gating. Br J Haematol. 2000;111(4):1198–1208. 10.1046/j.1365-2141.2000.02433.x 11167762

[7] Sherman GG, Galpin JS, Patel JM, Mendelow BV, Glencross DK CD4+ T cell enumeration in HIV infection with limited resources. J Immunol Methods. 1999;222(1–2):209–217. 10.1016/S0022-1759(98)00172-0 10022387

[8] Janossy G, Jani IV, Bradley NJ, Bikoue A, Pitfield T, Glencross DK Affordable CD4(+)-T-cell counting by flow cytometry: CD45 gating for volumetric analysis. Clin Diagn Lab Immunol. 2002;9(5):1085–1094. 10.1128/CDLI.9.5.1085-1094.2002 12204964 PMC120051

[9] Glencross DK, Coetzee LM, Faal M, et al PIMA™ CD4 counting accuracy depends on rigid finger-prick protocol adherence. 2011; 5th SA AIDS Conference, Durban, Jun 7–10.

[10] Jani IV, Sitoe NE, Chongo PL, et al Accurate CD4 T-cell enumeration and antiretroviral drug toxicity monitoring in primary healthcare clinics using point-of-care testing. AIDS. 2011;25(6):807–812. 10.1097/QAD.0b013e328344f424 21378535

[11] Glencross DK, Coetzee LM, Cassim N An Integrated Tiered Service Delivery Model (ITSDM) based on local CD4 testing demands can improve turn-around times and save costs whilst ensuring accessible and scalable CD4 services across a national programme. PLoS One. 2014;9(12):e114727 10.1371/journal.pone.0114727 25490718 PMC4260875

[12] Glencross DK, Janossy G, Coetzee LM, et al Large-scale affordable PanLeucogated CD4+ testing with proactive internal and external quality assessment: In support of the South African national comprehensive care, treatment and management programme for HIV and AIDS. Cytometry B Clin Cytom. 2008;74 Suppl 1:S40–S51. 10.1002/cyto.b.20384 18228554

[13] Glencross DK, Mendelow BV, Stevens WS Laboratory monitoring of HIV/AIDS in a resource-poor setting. S Afr Med J. 2003;93(4):262–263. 12806710

[14] Glencross DK, Aggett HM, Stevens WS, Mandy F African regional external quality assessment for CD4 T-cell enumeration: Development, outcomes, and performance of laboratories. Cytometry B Clin Cytom. 2008;74 Suppl 1:S69–S79. 10.1002/cyto.b.20397 18228560

[15] Pattanapanyasat K, Shain H, Noulsri E, et al A multicenter evaluation of the PanLeucogating method and the use of generic monoclonal antibody reagents for CD4 enumeration in HIV-infected patients in Thailand. Cytometry B Clin Cytom. 2005;65(1):29–36. 10.1002/cyto.b.20052 15800883

[16] Glencross DK, Scott LE, Jani IV, Barnett D, Janossy G CD45-assisted PanLeucogating for accurate, cost-effective dual-platform CD4+ T-cell enumeration. Cytometry. 2002;50(2):69–77. 10.1002/cyto.10068 12116348

[17] Denny TN, Gelman R, Bergeron M, et al A North American multilaboratory study of CD4 counts using flow cytometric panLeukogating (PLG): A NIAID-DAIDS Immunology Quality Assessment Program Study. Cytometry B Clin Cytom. 2008;74 Suppl 1:S52–S64. 10.1002/cyto.b.20417 18351622

[18] Cassim N, Coetzee LM, Schnippel K, Glencross DK Estimating implementation and operational costs of an integrated tiered CD4 service including laboratory and point of care testing in a remote health district in South Africa. PLoS One. 2014;9(12):e115420 10.1371/journal.pone.0115420 25517412 PMC4269438

[19] Coetzee LM, Cassim N, Glencross DK Implementation of a new ‘community’ laboratory CD4 service in a rural health district in South Africa extends laboratory services and substantially improves local reporting turnaround time. S Afr Med J. 2015;106(1):82–87. 10.7196/SAMJ.2016.v106i1.10081 26792313

[20] Beckman Coulter AQUIOS tetra system guide. In: Performance characteristics. April 2015;pp 6-1 to 6-1, accessed 15 Dec 2018. Available from: https://www.beckmancoulter.com/wsrportal/techdocs?docname=B26364AB.pdf

[21] Jentsch-Ullrich K, Koenigsmann M, Mohren M, Franke A Lymphocyte subsets’ reference ranges in an age- and gender-balanced population of 100 healthy adults – A monocentric German study. Clin Immunol. 2005;116(2):192–197. 10.1016/j.clim.2005.03.020 15993366

[22] Santagostino A, Garbaccio G, Pistorio A, et al An Italian national multicenter study for the definition of reference ranges for normal values of peripheral blood lymphocyte subsets in healthy adults. Haematologica. 1999;84(6):499–504. 10366792

[23] Moreno-Galvan M, Palafox A CD4+ CD8+ T cell reference values in the Mexico City population. Clin Vac Immunol. 2013;20(2):306–308. 10.1128/CVI.00523-12 PMC3571261 23239806

[24] Lawrie D, Coetzee LM, Becker P, Mahlangu J, Stevens W, Glencross DK Local reference ranges for full blood count and CD4 lymphocyte count testing. S Afr Med J. 2009;99(4):243–248. 19588777

[25] Bussmann H, Wester CW, Masupu KV, et al Low CD4+ T-lymphocyte values in human immunodeficiency virus-negative adults in Botswana. Clin Diagn Lab Immunol. 2004;11(5):930–935. 10.1128/CDLI.11.5.930-935.2004 15358655 PMC515279

[26] Crampin AC, Mwaungulu FD, Ambrose LR, Longwe H, French N Normal range of CD4 cell counts and temporal changes in two HIV negative Malawian populations. Open AIDS J. 2011;5:74–79. 10.2174/1874613601105010074 21892376 PMC3162193

[27] Ngowi BJ, Mfinanga SG, Bruun JN, Morkve O Immunohaematological reference values in human immunodeficiency virus-negative adolescent and adults in rural northern Tanzania. BMC Infect Dis. 2009;9:1 10.1186/1471-2334-9-1 19144106 PMC2630915

[28] Saathoff E, Schneider P, Kleinfeldt V, et al Laboratory reference values for healthy adults from southern Tanzania. Trop Med Int Health. 2008;13(5):612–625. 10.1111/j.1365-3156.2008.02047.x 18331386

[29] Urassa WK, Mbena EM, Swai AB, Gaines H, Mhalu FS, Biberfeld G Lymphocyte subset enumeration in HIV seronegative and HIV-1 seropositive adults in Dar es Salaam, Tanzania: Determination of reference values in males and females and comparison of two flow cytometric methods. J Immunol Methods. 2003;277(1–2):65–74. 10.1016/S0022-1759(03)00174-1 12799040

[30] Bosire EM, Nyamache AK, Gicheru MM, Khamadi SA, Lihana RW, Okoth V Population specific reference ranges of CD3, CD4 and CD8 lymphocyte subsets among healthy Kenyans. AIDS Res Ther. 2013;10(1):24 10.1186/1742-6405-10-24 24199645 PMC3827884

[31] Kibaya RS, Bautista CT, Sawe FK, et al Reference ranges for the clinical laboratory derived from a rural population in Kericho, Kenya. PLoS One. 2008;3(10):e3327 10.1371/journal.pone.0003327 18833329 PMC2553265

[32] Karita E, Ketter N, Price MA, et al CLSI-derived hematology and biochemistry reference intervals for healthy adults in eastern and southern Africa. PLoS One. 2009;4(2):e4401 10.1371/journal.pone.0004401 19197365 PMC2632744

[33] Nanzigu S, Waako P, Petzold M, et al CD4-T-lymphocyte reference ranges in Uganda and its influencing factors. Labmedicine. 2011;42(2):94–100. 10.1309/LMFT0VCE1UGO9YGD

[34] Gize A, Mathewos B, Moges B, Workineh M, Gedefaw L Establishment of normal reference intervals for CD3(+), CD4(+), CD8(+), and CD4(+) to CD8(+) ratio of T lymphocytes in HIV negative adults from University of Gondar Hospital, North West Ethiopia. AIDS Res Treat. 2014;2014:267450 10.1155/2014/267450 25485147 PMC4251638

[35] Tsegaye A, Messele T, Tilahun T, et al Immunohematological reference ranges for adult Ethiopians. Clin Diagn Lab Immunol. 1999;6(3):410–414. 10225845 10.1128/cdli.6.3.410-414.1999 PMC103732

[36] Oladepo DK, Idigbe EO, Audu RA, et al Establishment of reference values of CD4 and CD8 lymphocyte subsets in healthy Nigerian adults. Clin Vaccine Immunol. 2009;16(9):1374–1377. 10.1128/CVI.00378-08 19641097 PMC2745013

[37] Klose N, Coulibaly B, Tebit DM, et al Immunohematological reference values for healthy adults in Burkina Faso. Clin Vaccine Immunol. 2007;14(6):782–784. 10.1128/CVI.00044-07 17442846 PMC1951086

[38] Murugavel KG, Balakrishnan P, Mohanakrishnan J, et al Establishment of T-lymphocyte subset reference intervals in a healthy adult population in Chennai, India. Indian J Med Res. 2009;129(1):59–63. 19287058

[39] Uppal SS, Verma S, Dhot PS Normal values of CD4 and CD8 lymphocyte subsets in healthy Indian adults and the effects of sex, age, ethnicity, and smoking. Cytometry B Clin Cytom. 2003;52(1):32–36. 10.1002/cyto.b.10011 12599179

[40] Chng WJ, Tan GB, Kuperan P Establishment of adult peripheral blood lymphocyte subset reference range for an Asian population by single-platform flow cytometry: Influence of age, sex, and race and comparison with other published studies. Clin Diagn Lab Immunol. 2004;11(1):168–173. 10.1128/CDLI.11.1.168-173.2004 14715565 PMC321350

[41] Wong WS, Lo AW, Siu LP, et al Reference ranges for lymphocyte subsets among healthy Hong Kong Chinese adults by single-platform flow cytometry. Clin Vaccine Immunol. 2013;20(4):602–606. 10.1128/CVI.00476-12 23408529 PMC3623424

[42] Jiang W, Kang L, Lu HZ, et al Normal values for CD4 and CD8 lymphocyte subsets in healthy Chinese adults from Shanghai. Clin Diagn Lab Immunol. 2004;11(4):811–813. 10.1128/CDLI.11.4.811-813.2004 15242966 PMC440627

[43] Clinical Laboratory Standards Institute (CLSI) Method comparison and bias estimation using patient samples. 2nd ed. CLSI guideline EP09-A2 Wayne, PA: CLSI; 2002.

[44] Clinical Laboratory Standards Institute (CLSI) Defining, establishing, and verifying reference intervals in the clinical laboratory. 3rd ed. CLSI guideline C28-A3 Wayne, PA: CLSI; 2010.

[45] Bland JM, Altman DG Statistical methods for assessing agreement between two methods of clinical measurement. Lancet. 1986;327(8476):307–310. 10.1016/S0140-6736(86)90837-8 2868172

[46] Bwana P, Vojnov L, Adhiambo M, et al The BD FACS presto point of care CD4 test accurately enumerates CD4+ T cell counts. PLoS One. 2015;10(12):e0145586 10.137/journal.pone.0145586 26720601 PMC4697849

[47] Scott LE, Galpin JS, Glencross DK Multiple method comparison: Statistical model using percentage similarity. Cytometry B Clin Cytom. 2003;54B(1):46–53. 10.1002/cyto.b.10016 12827667

[48] Coetzee LM, Glencross DK Performance verification of the new fully automated Aquios flow cytometer PanLeucogate (PLG) platform for CD4-T-lympocyte enumeration in South Africa. PLoS One. 2017;12(11):e0187456 10.1371/journal.pone.0187456 29099874 PMC5669480

[49] Comans-Bitter WM, De Groot R, Van Den Beemd R, et al Immunophenotyping of blood lymphocytes in childhood. Reference values for lymphocyte subpopulations. J Pediatr. 1997;130(3):388–393. 10.1016/S0022-3476(97)70200-2 9063413

[50] Peeling RW, Sollis KA, Glover S, et al CD4 enumeration technologies: A systematic review of test performance for determining eligibility for antiretroviral therapy. PLoS One. 2015;10(3):e0115019 10.1371/journal.pone.0115019 25790185 PMC4366094

[51] Stevens W, Gelman R, Glencross DK, Scott LE, Crowe SM, Spira T Evaluating new CD4 enumeration technologies for resource-constrained countries. Nat Rev Microbiol. 2008;6:S29–S38. 10.1038/nrmicro2000 22745957

[52] World Health Organization Consolidated guidelines on the use of antiretroviral drugs for treating and preventing HIV infection: Recommendations for a public health approach. Geneva, Switzerland: WHO; 2016. 27466667

[53] Mandala WL, Ananworanich J, Apornpong T, et al Control lymphocyte subsets: Can one country’s values serve for another’s? J Allergy Clin Immunol. 2014;134(3):759–761.e8. 10.1016/j.jaci.2014.06.030 25171870 PMC4150016

[54] Maini MK, Gilson RJ, Chavda N, et al Reference ranges and sources of variability of CD4 counts in HIV-seronegative women and men. Genitourin Med. 1996;72(1):27–31. 10.1136/sti.72.1.27 8655163 PMC1195587

[55] Glencross DK, Stevens G, Scott LE, Mendelow BV, Stevens W The challenge of laboratory monitoring of HIV. S Afr Med J. 2002;92(4):248. 12056341

[56] Scott LE, Glencross DK Monitoring reproducibility of single analysis, single platform CD4 cell counts in a high volume, low resource laboratory setting using sequential bead count rates. Cytometry B Clin Cytom. 2005;67(1):31–32. 10.1002/cyto.b.20066 16100713

[57] Denny T, Gelman R, Bergeron M, et al A multi-lab study of CD4 counts using flow cytometric PanLeukogating (PLG): A NIAID-DAIDS Immunology Quality Assessment Program study. CROI-Conference on Retrovirus and Opportunistic Infections Boston, MA; 2005.

[58] Zhang J, Quessada V, De Castro J An automated cell preparation method for flow CARETM Pan-Leukocyte Gating (PLG) CD4 assay in a 96-well plate offers increased throughput and ease of use (Poster) African Society for Laboratory Medicine (ASLM) Cape Town, South Africa; 2016.

[59] World Health Organization (WHO) Emergency scale-up of antiretroviral therapy in resource-limited settings: Technical and operational recommendations to achieve 3 by 5 [homepage on the Internet]. Zambia; 2003 Nov 18–21 [cited 2018 Dec 15]. Available from: https://www.who.int/3by5/publications/documents/en/zambiaen.pdf

[60] Ford N, Meintjes G, Pozniak A, et al The future role of CD4 cell count for monitoring antiretroviral therapy. Lancet Infect Dis. 2015;15(2):241–247. 10.1016/S1473-3099(14)70896-5 25467647

[61] Carmona S, Bor J, Nattey C, et al Persistent high burden of advanced HIV disease among patients seeking care in South Africa’s National HIV Program: Data from a nationwide laboratory cohort. Clin Inf Dis. 2018;66 Suppl 2:S111–S117. 10.1093/cid/ciy045 PMC5850436 29514238

[62] Coetzee LM, Cassim N, Glencross DK Analysis of HIV disease burden by calculating the percentages of patients with CD4 counts <100 cells/microl. Across 52 districts reveals hot spots for intensified commitment to programmatic support. S Afr Med J. 2017;107(6):507–513. 10.7196/SAMJ.2017.v107i6.11311 28604323

[63] Guidelines for managing advance HIV disease and rapid initiation of antiretroviral therapy, July 2017 [homepage on the Internet]. Geneva: World Health Organization; 2017 [cited 2018 Dec 15]. Available from: https://www.who.int/hiv/pub/guidelines/advanced-HIV-disease/en/29341560

[64] National consolidated guidelines for the prevention of mother-to-child transmission of HIV (PMTCT) and the management of HIV in children, adolescents and adults [homepage on the Internet]. Pretoria, South Africa: Department of Health, Republic of South Africa; 2015 [cited 2018 Dec 15]. Available from: https://sahivsoc.org/Files/ART%20Guidelines%2015052015.pdf

[65] Ford N, Meintjes G, Vitoria M, Greene G, Chiller T The evolving role of CD4 cell counts in HIV care. Curr Opin HIV AIDS. 2017;12(2):123–128. 10.1097/COH.0000000000000348 28059957

[66] Moorhouse M, Conradie F, Venter F What is the role of CD4 count in a large public health antiretroviral programme? S Afr J HIV Med. 2016;17(1):446 10.4102/sajhivmed.v17i1.446 PMC5843041 29568607

